# Sequence Features of *E. coli* mRNAs Affect Their Degradation

**DOI:** 10.1371/journal.pone.0028544

**Published:** 2011-12-07

**Authors:** Gal Lenz, Adi Doron-Faigenboim, Eliora Z. Ron, Tamir Tuller, Uri Gophna

**Affiliations:** 1 Department of Molecular Microbiology and Biotechnology, George S. Wise Faculty of Life Sciences, Tel Aviv University, Ramat Aviv, Israel; 2 Iby and Aladar Fleischman Faculty of Engineering, Tel Aviv University, Ramat Aviv, Israel; University of Wyoming, United States of America

## Abstract

Degradation of mRNA in bacteria is a regulatory mechanism, providing an efficient way to fine-tune protein abundance in response to environmental changes. While the mechanisms responsible for initiation and subsequent propagation of mRNA degradation are well studied, the mRNA features that affect its stability are yet to be elucidated. We calculated three properties for each mRNA in the *E. coli* transcriptome: G+C content, tRNA adaptation index (tAI) and folding energy. Each of these properties were then correlated with the experimental transcript half life measured for each transcript and detected significant correlations. A sliding window analysis identified the regions that displayed the maximal signal. The correlation between transcript half life and both G+C content and folding energy was strongest at the 5′ termini of the mRNAs. Partial correlations showed that each of the parameters contributes separately to mRNA half life. Notably, mRNAs of recently-acquired genes in the *E. coli* genome, which have a distinct nucleotide composition, tend to be highly stable. This high stability may aid the evolutionary fixation of horizontally acquired genes.

## Introduction

The capacity to selectively degrade transcripts of different genes at different rates enhances the capability of bacteria to regulate their proteome in response to environmental changes [1], [2], [3]. Although bacterial transcripts are generally less stable than eukaryotic mRNAs, inter-gene variation within the same transcriptome can be very high. Thus, in *Escherichia coli,* the half lives of transcripts range from less than a minute to more than half an hour [4], [5]. Although the mechanism for mRNA degradation in Gram-negative bacteria has been extensively studied in recent years, and the enzymes that are involved in transcript degradation have been well characterized (For a review see [6]), the sequence features that determine the eventual mRNA half lives are yet to be elucidated. Previous studies showed that mRNAs encoding distinct protein functions can differ in their respective mRNA stability [4] and that longer mRNAs in *E. coli* are generally less stable [7].

Mechanistically, degradation of mRNA is influenced not only by the degradosome complex and other RNAses [6], but can also be regulated through binding of RNA chaperons. These small nucleic acid-binding proteins have been shown to increase the half lives of their target transcripts through direct binding, resulting in changes of the secondary structure of the mRNA. In *E. coli* Hfq [8], [9]and the cold shock family of proteins [10], [11], [12]are the best studied examples of RNA chaperons, and were all shown to preferably bind A+T rich mRNA sequences. It therefore follows that the sequence nucleotide composition or folding energy can play a role in determining the half life of a transcript [13]. Here we examine three characteristics of mRNAs, G+C content, tRNA adaptation index (tAI) and folding energy. We find these parameters to be significantly correlated with transcript half-life, and identify the key regions in the nucleotide sequence that affect this property.

## Materials and Methods

### Calculation of sequence properties

Calculation of tAI was preformed as described in [14] using the equation described in [15]. Folding energy was calculated using UNAfold [16], with a 40-nucleotide long sliding window, with a step size of 1 nucleotide, starting 40-nucleotides before the start codon and ending at the end of the ORF. G+C content was also calculated using a 40-nucleotide long sliding window, with a step size of 1 nucleotide for every transcript within the *E. coli* transcriptome beginning at nucleotide -50 before the start codon.

### Statistical analysis

All statistical analyses were performed using SPSS 15 for windows (IBM, USA). Non-parametric methods, such as the Kruskal-Wallis test and Spearman correlations, were used as these analyses require no assumptions on the distribution of the data.

### Data set normalization

All decay rate values were divided by the mean half life of each data set to create a distance from average (DFA) value. DFA values from each data set were divided by their equivalent from the other data set to determine the degree of change under the different growth conditions. We used only transcripts with a degree of change of 20% or less between the two experiments, thus avoiding genes that experienced physiological regulatory changes between experiments, and creating a more reliable dataset of mRNA decay rates.

## Results and Discussion

### 5′ sequence characteristics influence mRNAs stability in E. coli

**Figure 1:**
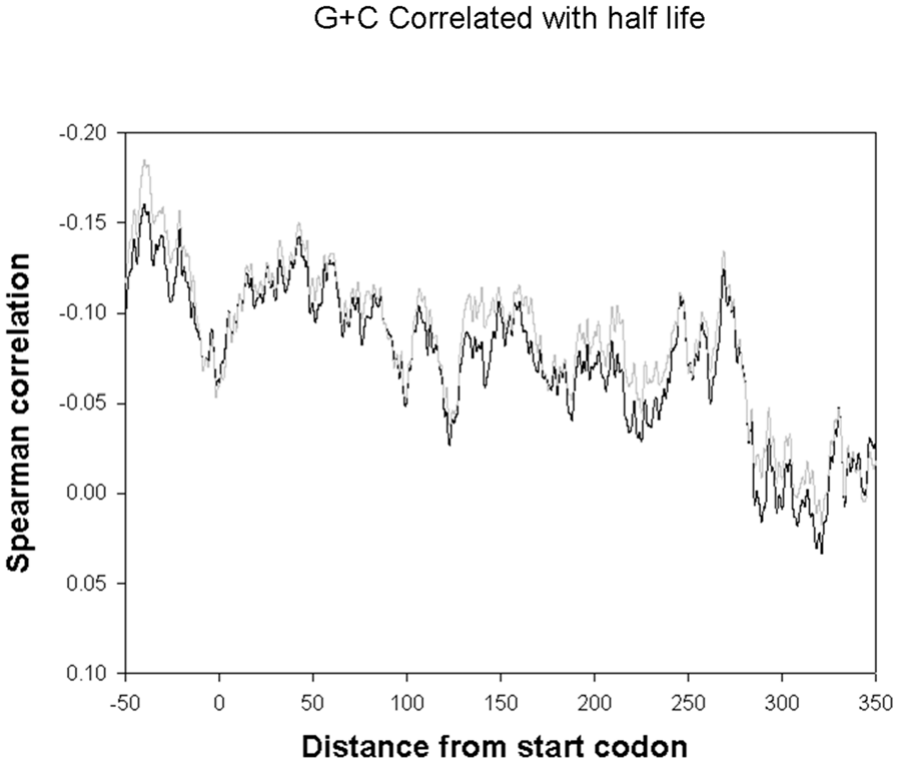
A sliding window analysis of the correlation between the *E. coli* transcript G+C content and its respective half-life in the two data sets. The strongest correlated window starts at nucleotide -40 (Spearman R = −0.185, p = 0.002 and R = −0.16 p = 0.003 respectively).

In order to examine whether there is a global trend that links G+C content and the stability of mRNAs in *E. coli*, we first calculated the G+C content of every transcript in the *E. coli* transcriptome. Subsequently, we correlated G+C content data with experimental half life values obtained for cells grown on a minimal medium (M9), and a rich medium (LB) in 30°C from two separate experiments (Fig. S1). [5], these data were acquired using two-color fluorescent microarrays and validated using a northern blot analysis. A moderate but significant correlation was obtained between G+C and mRNA half-life (Spearman rank correlations of R = −0.172 and R = −0.130 for minimal and rich media, respectively, p<0.0001 for both analyses). Since mRNA decay rate for some genes may differ across the growth conditions tested, due to physiological regulation, we normalized the half-life of each transcript in the two data sets (see Materials and Methods) and repeated our analysis, this time using only transcripts whose normalized half-lives remained similar under both experimental conditions (See Materials and Methods). Out of 2042 genes with measured half lives in both media, we thus proceeded using 687 genes that satisfied this criterion. Filtering out media-specific trends resulted in stronger correlations (R = −0.181 and R = −0.194 for minimal and rich media, respectively, p<0.0001). To investigate whether this correlation is derived from a global genome-wide trend or stems from extreme cases only, we performed the same correlation analysis, this time leaving out both the 10% least stable and the 10% most stable mRNAs. Excluding these extreme cases had little effect on the resulting correlation (R = −0.171 and R = −0.188 for minimal and rich media, respectively, p<0.0001). A 40-nucleotide long sliding window was then applied, in order to locate the specific regions within the mRNA where G+C content has the strongest effect on the respective half-life (Fig. 1). The strongest correlated window was found to begin at nucleotide -40 from the ORF's start codon (Spearman correlation R = −0.185 and R = −0.160 for minimal and rich media, respectively, p<0.0001), and this correlation decayed toward the 3′ end of the transcript (R<0.02). It was experimentally shown that the 5′ of transcripts can be actively degraded[17] and that A+U rich regions in 5′ UTR could result in enhanced stability and a better translation of the *lacZ* mRNA [18]. Our analysis implies that this observation may reflect a global trend in *E. coli*, since A+T rich regions in 5′ UTR are correlated with a reduced decay rate in its entire transcriptome.

**Figure 2:**
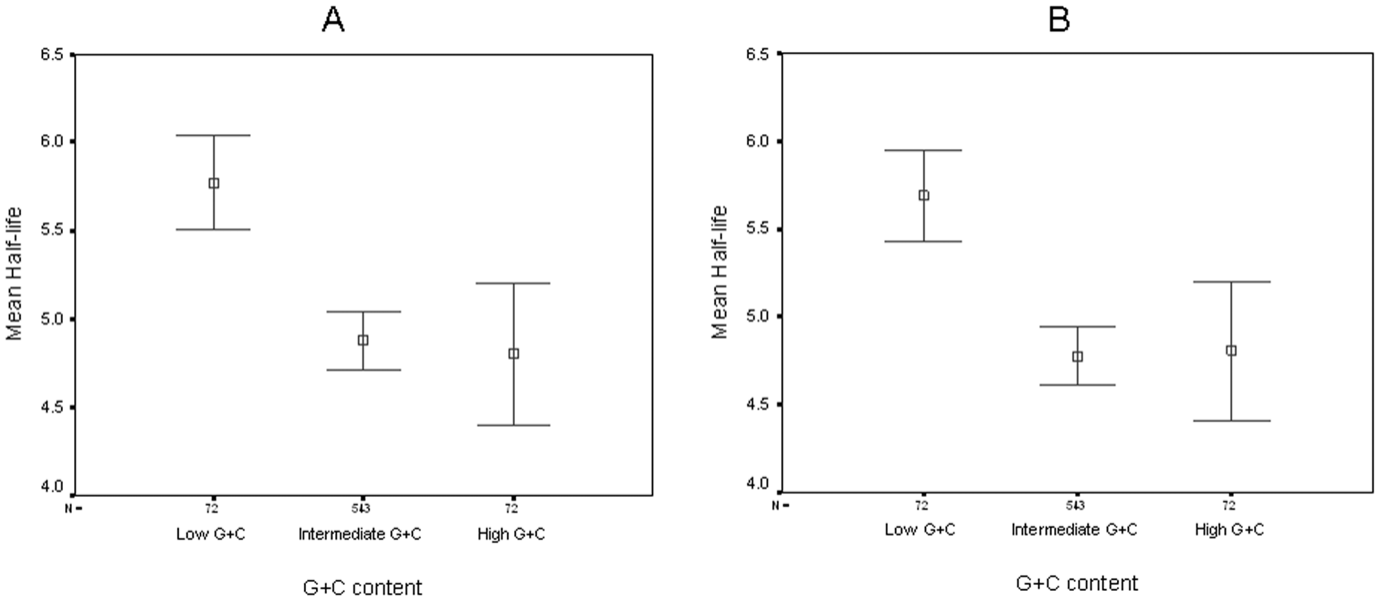
Average half lives of *E. coli* transcripts binned according to G+C content. The number of genes (N) is listed above each group's designation. Vertical bars represent 95% confidence intervals. Boxes represent the mean of the group. A- M9 media; B- LB media.

To further establish the impact of G+C content on mRNA stability, we divided the *E. coli* genome into three bins of genes according to their G+C content. The three groups were chosen so that the high G+C (>0.55) and low G+C (<0.4) bins had a similar number of genes, while the rest of genome (0.4<G+C<0.55) provided a reference group with intermediate G+C content. We observed that genes with a low G+C content were on average markedly more stable than the rest of the transcripts (mean half life 5.68 and 5.77 minutes, median half life 5.6 and 5.8 minutes for minimal and rich media, respectively), while the intermediate and high G+C bins showed no significant difference (mean half life 4.77 and 4.87 minutes, median half life 4.6 and 4.7 minutes for minimal and rich media, respectively for the intermediate and mean half life of 4.8 and 4.81 minutes, median half life of 4.55 and 4.75 for the High G+C bins, see Figure 2). The difference between the low G+C group and both the intermediate and high G+C groups was statistically significant (p = 1.46*10^−8^, Kruskal-Wallis test), while the difference between the intermediate and high G+C was not (p = 0.965 Kruskal-Wallis test).

**Figure 3:**
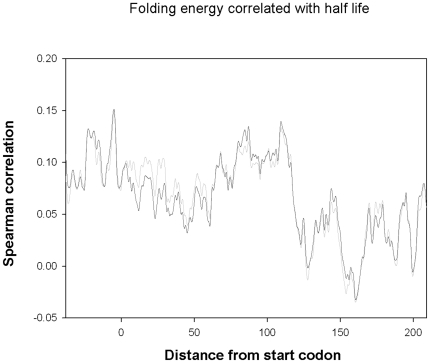
A sliding window analysis of the correlation between the folding energy of *E. coli* transcripts and their respective half-life in each data set. The strongest correlated window starts at nucleotide -5 (Spearman R = 0.150, p = 0.00015 and R = 0.151, p = 0.00014 respectively).

The correlation between the G+C content and mRNA stability could be due to mRNA structure or to other physiological factors such as binding of stabilizing proteins or the presence of digestion sites for RNases. To distinguish between these alternatives we correlated the folding energies of mRNAs with their respective half lives (Fig. S2). Using a 40-nucleotide long sliding window we observed a significant correlation (Fig. 3), where the strongest correlated window started at nucleotide -5 for both data sets (R = 0.15 for both minimal and rich media) indicating that transcripts with less stable secondary structures in their 5′ ends tend to be less sensitive to degradation. Jia and Li [13] previously showed that less stable secondary structures results in increased half life in cells grown on rich media. We suggest this phenomenon is global, and also pin point the location of the stability-determining region to the 5′ region of transcripts. Furthermore, partial correlations and a co-linearity tests showed that G+C content and folding energy both have a distinct contribution to mRNA half life (Tables 1 and 2, for pairwise correlations between the parameters see table S1).

**Table 1:** *E. coli* parameters correlated with half life (LB) using partial correlation.

G+C controlled for Folding	R = −0.1338	P<0.001
G+C controlled for tAI	R = −0.1175	P<0.001
tAI controlled for G+C	R = −0.1297	P<0.001

**Table 2:** *E. coli* parameters correlated with half life (M9) using partial correlation.

G+C controlled for Folding	R = −0.1465	P<0.001
G+C controlled for tAI	R = −0.1232	P<0.001
tAI controlled for G+C	R = −0.1411	P<0.001

It is established that the 5′ regions of transcripts differ from the rest of the mRNAs in both folding energy and codon usage [19], [20], [21]}. We show both G+C content and folding energy to be distinctly lower in the 5′ region of transcripts (figures S3 and S4, respectively). Whether the different codon usage in this region is simply the result of selection for reduced mRNA secondary structure [19], [21] or is in place to slow down initial translation thus allowing better spacing of ribosomes [20], [22] , is still under dispute.

These findings suggest a meaningful physiological contribution of sequence to the mRNA's ensuing decay rate. It is improbable that this correlation results from less optimal digestion sites for RNAses, since it is well established that the rate limiting step in mRNA degradation in *E. coli* is the initial endonucleolytic digestion by RNase E, which in fact **prefers** A+T rich sequences[1], [23], rather than vice versa. A more likely explanation is a protective effect of RNA-binding proteins such as RNA chaperons [2], [24], which are known to bind A+T rich regions, or a balance between chaperones and RNases that leans toward the former. Nevertheless, the correlations we observed do not necessarily indicate a causal relationship and could result from additional molecular mechanisms that remain to be discovered.

### Transcripts that contain less efficient codons are more stable

The tRNA Adaptation Index (tAI) defines the codon efficiency of mRNA in relation to the inferred abundance of tRNA in the cell [25]. A higher tAI should therefore decrease ribosome stalling and increase the rate of translation. During its translation, an mRNA bound by the ribosome is thought to be less prone to degradation, since the translation machinery protects it from the degradozome [26], [27]. We tested whether there is a correlation between the codon optimality and the half-life of the transcript. We therefore calculated the tAI values for the entire transcriptome of *E. coli* and correlated them with experimental half-lives obtained in minimal and rich media (Fig. S5), as described above [5]. We found a significant negative correlation between the two parameters (Spearman correlation, R = −0.189 and R = −0.203 for minimal and rich media respectively p<0.001). As tAI and G+C content are not totally independent features (i.e. less efficient codons tend to have atypical G+C content) we used partial correlations, and observed each parameter to contribute separately to stability (Table 1).

**Figure 4:**
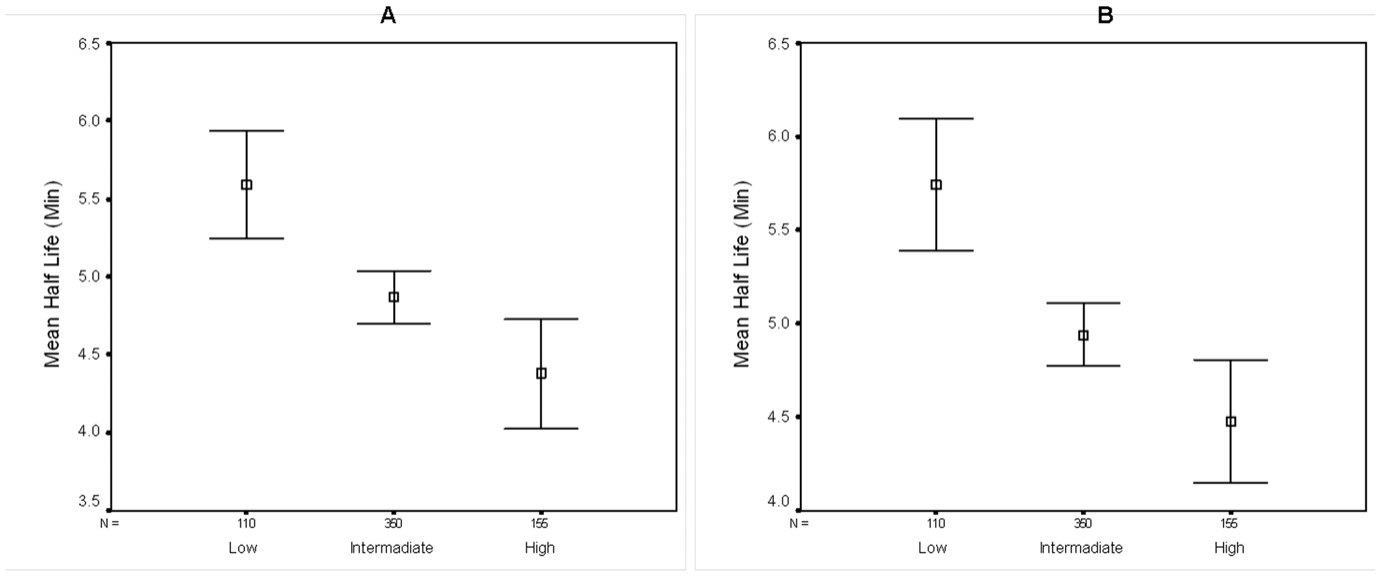
Effect of tAI on transcript stability. The normalized *E. coli* dataset was divided into 3 subsets of genes according to their tAI. The number of genes (N) is listed above each group's designation. Vertical bars represent 95% confidence intervals. Boxes represent the mean half life of the group. A- M9 media; B- LB media.

We subsequently divided the normalized half-life dataset (see above) into three subsets of genes according to their tAI values with both the low (tAI<0.22) and high (tAI<0.266) groups having similar gene counts, using the intermediate group (0.22<tAI<0.26) as a reference (Fig. 4). Generally, genes with a relatively low tAI have transcripts that are markedly more stable than genes with intermediate tAI, which are in turn more stable then transcripts of genes with a high tAI value (mean half life of 5.59 and 5.73 minutes, median half life of 5.4 and 5.65 minutes, for low tAI in minimal and rich media respectively; mean half life of 4.87 and 4.94 minutes, median half life of 4.7 and 4.9 minutes for intermediate tAI in minimal and rich media respectively; mean half life of 4.37 and 4.47 minutes, median half life of 4 and 4 minutes for high tAI in minimal and rich media respectively, p<0.0001, Kruskal-Wallis test).

It is tempting to suggest that sub-optimal codons, presumably leading to a longer time of translation and a longer association with the ribosome can protect the mRNA from degradation. Indeed, it was suggested by Kolmsee *et al* [23] that sub-optimal codons could play a positive role in the expression of sigma S in part due to increased mRNA stability. In view of our data we suggest that this observation could in fact represent a global phenomenon where sub-optimal codons contribute to increased stability of transcripts. Conversely, highly efficient translation will minimize ribosome association time and thereby decrease protection from degradation. However, one should keep in mind that at least for some genes, efficient codons are accompanied by a high translation initiation rate, and therefore the density of ribosomes along the mRNA can actually be higher in highly translated genes. To further test if proteins encoded less efficiently (having lower tAI), which are presumed to be less abundant, indeed have more stable mRNAs, we used recent *E. coli* protein abundance data obtained at the single cell resolution [28], and correlated the protein levels with the normalized half-life values. We observed a significant negative correlation between mRNA half-life and protein abundance (Spearman correlation, R = −0.169, p<0.001), indicating that the more abundant proteins do tend to correspond to less stable mRNAs. Clearly, the usage of optimal codons in highly expressed genes outweighs the reduced stability of their transcripts, or the net effect would not be high protein levels. Why do highly translated genes suffer more from transcript degradation in the first place, remains to be determined. One may speculate that increased translation could somehow “erode” the mRNA either directly, or by recruitment of RNases. Alternatively, the stalled ribosome could interact with RNA chaperons such as CspC, that have been shown to interact with a number of ribosomal proteins [29], thereby contributing to the stabilization of the translated mRNA.

Dos Reis and coworkers previously showed that a subset of genes with highly sub-optimal codons and high A+T content possessed a negative correlation between codon bias optimality and mRNA levels [25]. When we analyzed this particular subset of *E. coli* genes, we found that as a group they encode markedly more stable transcripts than those of the other subsets in that study, with average and high codon optimality (Mean half life of 6.14 minutes as compared to 4.8 and 4.3 minutes, median half life of 5.85 minutes as compared to 4.5 and 3.6 minutes for intermediate and high bias toward optimal codons, respectively p<0.001). Thus, differences in mRNA stability could help explain the surprising finding of increased mRNA levels within this high A+T content group [25]. Our analysis also shows that genes within this group have higher average protein abundance (according to ref [28])than those of genes described in [25] as having intermediate codon optimality (58.4 compared to 15.8 protein molecules per bacterial cell p<0.001).

### Laterally acquired genes in E. coli encode transcripts of increased stability compared to transcripts of native genes

**Figure 5:**
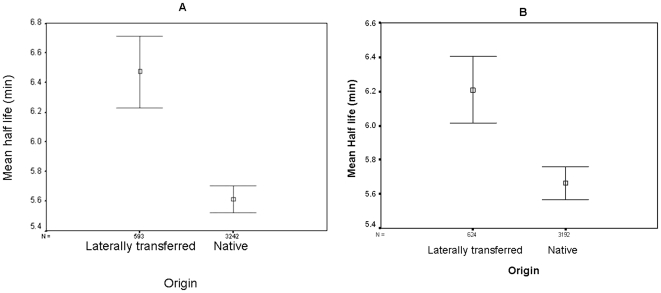
Difference in transcript stability between foreign and native genes. The *E. coli* genome was divided into two subsets of genes: recently transferred genes (LGT) (A: Genes described as recent LGT in [30], B: Genes described as LGT in [31], B) and non-recently transferred genes (Native). The number of genes (N) is listed above each group's designation. Vertical bars represent 95% confidence intervals. Boxes represent the mean of the group.

Interestingly, lower than average tAI (*i.e.* frequent use of suboptimal codons) and atypical G+C content are the hallmarks of genes recently acquired by *E. coli* through lateral gene transfer (LGT). Since we found both of these parameters to independently correlate with transcript half life, we examined whether transcripts of genes, inferred to have been recently transferred to *E. coli* by LGT, differ from all other transcripts in terms of their transcript stability. Indeed, transcripts of recently acquired genes, identified by two independent studies [30], [31], were found to be significantly more stable than those of non-recently acquired genes (having a mean half-life of 6.47 and 6.21 minutes, median half life of 6.1 and 5.9 minutes vs. mean half life of 5.61 and 5.64 minutes and median half life of 5.4 and 5.3 minutes of native genes in these datasets, p<0.0001, Kruskal-Wallis test, see figure 5). Acquiring new genes through LGT is an important engine of adaptation in bacteria. However, recently acquired genes will typically contain codons that are sub-optimal in their new host, and therefore will not be well-translated. Increased mRNA stability could offset some of the translational inadequacy by allowing more time for the translation of the message. Alternatively, slow translation due to inferior codons could simply result in a longer association with the ribosome and consequently, a more sustained protective effect.

In this study we indentified sequence determinants in transcripts that significantly correlate with their rates of decay. We have also found that transcripts of recently acquired genes in *E. coli* are markedly more stable then native genes, perhaps giving these genes a better chance to be expressed and thus contribute to bacterial fitness, and consequently become fixed in their new genomes.

## Supporting Information

Figure S1 **Scatter plot of G+C content against mRNA half life.**
(TIF)

Figure S2 **Scatter plot of Folding energy against mRNA half life.**
(TIF)

Figure S3 **Variation of average G+C content along the mRNA- G+C content was calculated using 40 nucleotide long sliding window for the entire** ***E. coli*** **transcriptome, Mean G+C was calculated for each window.**
(TIF)

Figure S4 **Variation of average folding energy along the mRNA.** Folding energy was calculated using a 40 nucleotide long sliding window for the entire *E. coli* transcriptome, Mean folding energy was calculated for each window.
(TIF)

Figure S5 **Scatter plot of tAI against mRNA half life.**
(TIF)

Table S1 **Correlations**
(DOC)
