# Supplementary figures and images for: Sequence Features of E. coli mRNAs Affect Their Degradation
Source: PLoS One. 2011 Dec 7;6(12):e28544. doi: 10.1371/journal.pone.0028544 (PMC3233582; doi:10.1371/journal.pone.0028544)

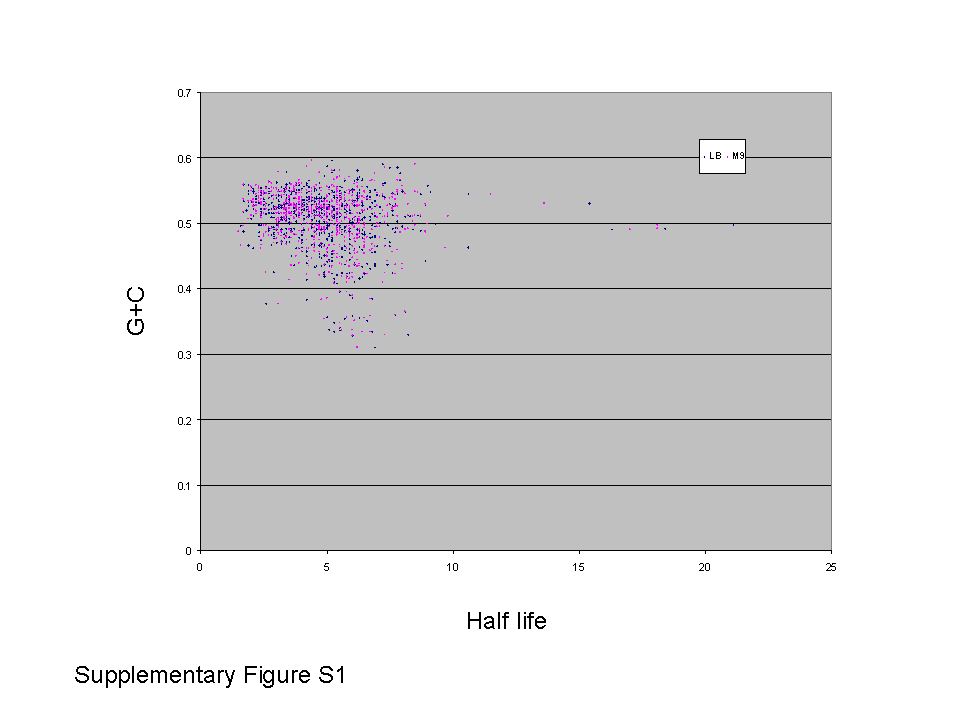

Supplement: Figure S1 — Scatter plot of G+C content against mRNA half life. (TIF) [file pone.0028544.s001.tif]

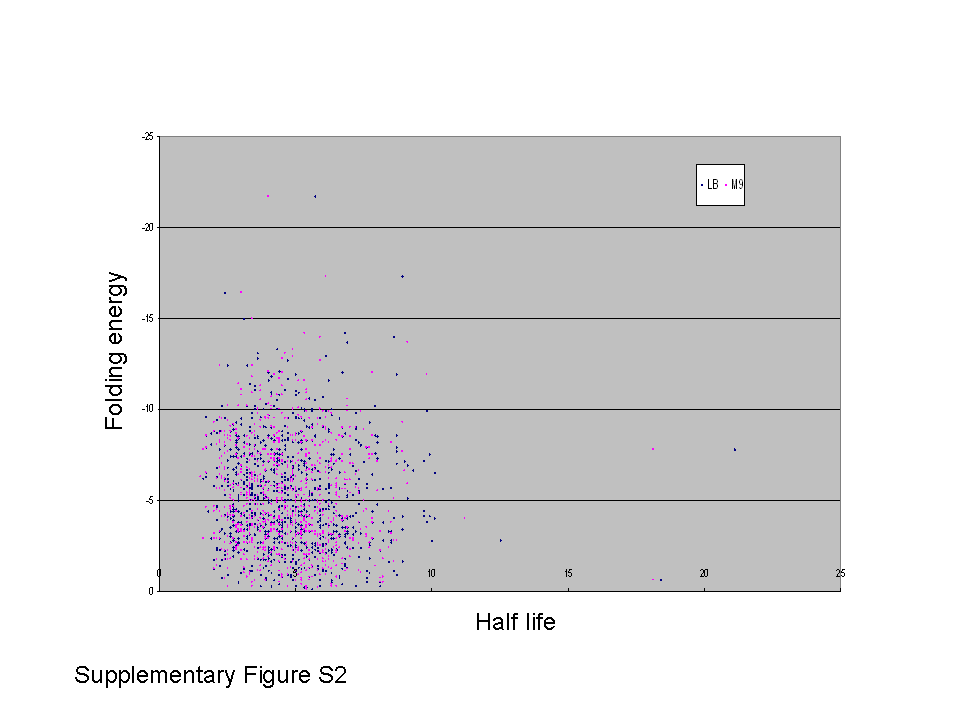

Supplement: Figure S2 — Scatter plot of Folding energy against mRNA half life. (TIF) [file pone.0028544.s002.tif]

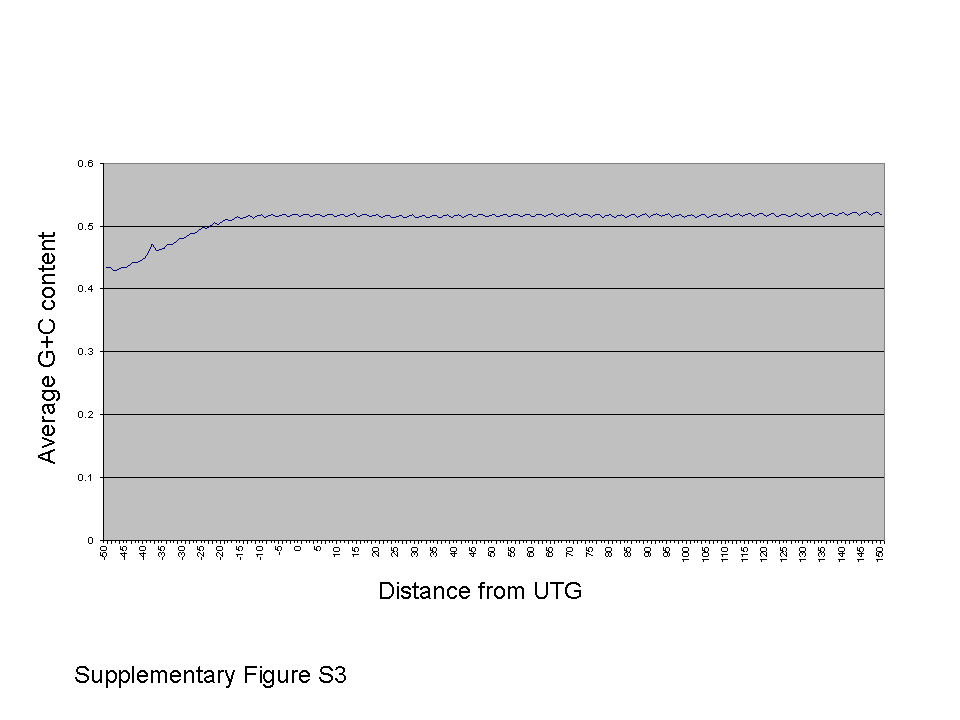

Supplement: Figure S3 — Variation of average G+C content along the mRNA- G+C content was calculated using 40 nucleotide long sliding window for the entire E. coli transcriptome, Mean G+C was calculated for each window. (TIF) [file pone.0028544.s003.tif]

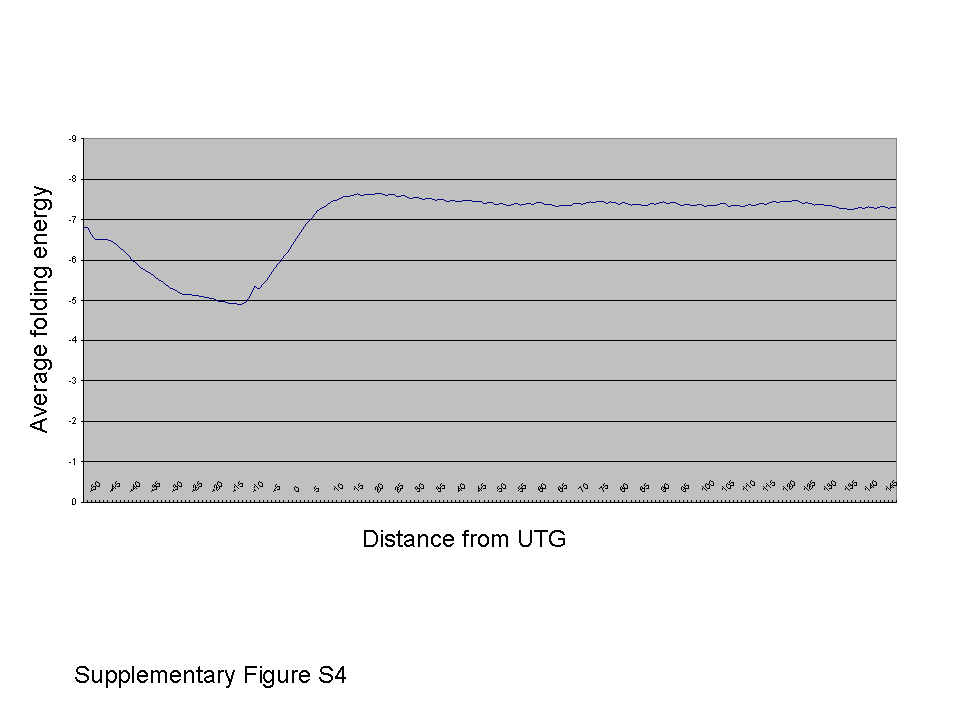

Supplement: Figure S4 — Variation of average folding energy along the mRNA. Folding energy was calculated using a 40 nucleotide long sliding window for the entire E. coli transcriptome, Mean folding energy was calculated for each window. (TIF) [file pone.0028544.s004.tif]

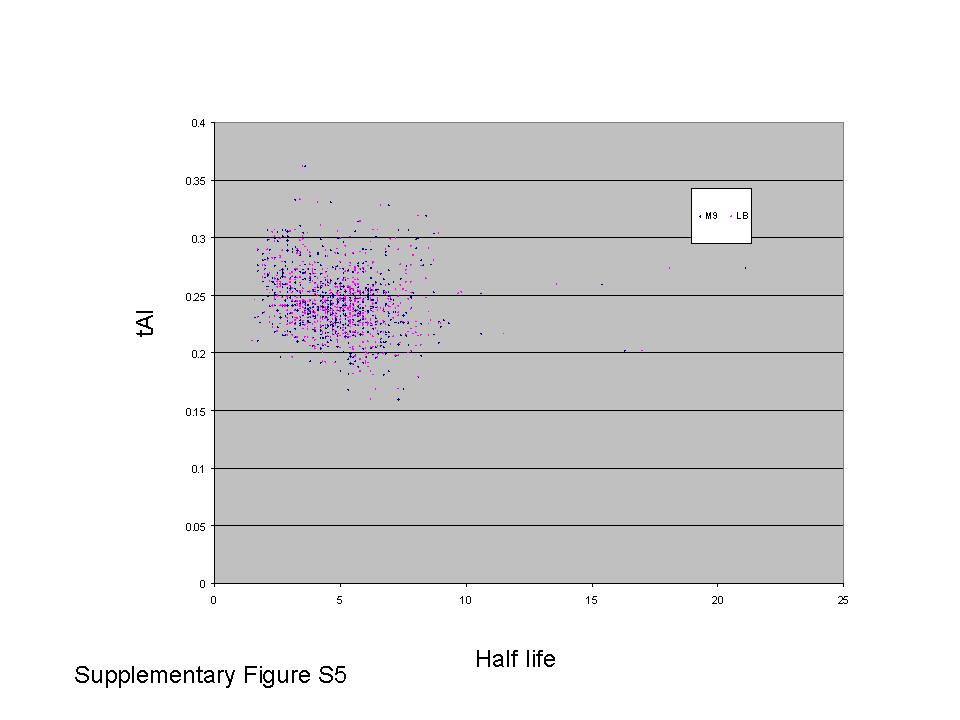

Supplement: Figure S5 — Scatter plot of tAI against mRNA half life. (TIF) [file pone.0028544.s005.tif]
