# Supplementary material for: Sequence Features of E. coli mRNAs Affect Their Degradation
Source: PLoS One. 2011 Dec 7;6(12):e28544. doi: 10.1371/journal.pone.0028544 (PMC3233582; doi:10.1371/journal.pone.0028544)
Supplement: Table S1 — Correlations (DOC) [file pone.0028544.s006.doc]

**Correlations**

|  |  |  | Length | G+C | LB Halflife | M9 halflife | tAI |
| --- | --- | --- | --- | --- | --- | --- | --- |
| Spearman's rho | Length | Correlation Coefficient | 1.000 | .431(**) | -.243(**) | -.247(**) | .131(**) |
|  |  | Sig. (2-tailed) | . | .000 | .000 | .000 | .001 |
|  |  | N | 687 | 687 | 687 | 687 | 619 |
|  | G+C | Correlation Coefficient | .431(**) | 1.000 | -.181(**) | -.194(**) | .125(**) |
|  |  | Sig. (2-tailed) | .000 | . | .000 | .000 | .002 |
|  |  | N | 687 | 687 | 687 | 687 | 619 |
|  | LB halflife | Correlation Coefficient | -.243(**) | -.181(**) | 1.000 | .940(**) | -.233(**) |
|  |  | Sig. (2-tailed) | .000 | .000 | . | .000 | .000 |
|  |  | N | 687 | 687 | 687 | 687 | 619 |
|  | M9 halflife | Correlation Coefficient | -.247(**) | -.194(**) | .940(**) | 1.000 | -.236(**) |
|  |  | Sig. (2-tailed) | .000 | .000 | .000 | . | .000 |
|  |  | N | 687 | 687 | 687 | 687 | 619 |
|  | tAI | Correlation Coefficient | .131(**) | .125(**) | -.233(**) | -.236(**) | 1.000 |
|  |  | Sig. (2-tailed) | .001 | .002 | .000 | .000 | . |
|  |  | N | 619 | 619 | 619 | 619 | 619 |

** Correlation is significant at the 0.01 level (2-tailed).
